# Validation of Selective Agars for Detection and Quantification of Escherichia coli Strains Resistant to Critically Important Antimicrobials

**DOI:** 10.1128/Spectrum.00664-21

**Published:** 2021-11-10

**Authors:** Zheng Z. Lee, Rebecca Abraham, Mark O’Dea, Ali Harb, Kelly Hunt, Terence Lee, Sam Abraham, David Jordan

**Affiliations:** a Antimicrobial Resistance and Infectious Diseases Laboratory, Murdoch University, Murdoch, Western Australia, Australia; b New South Wales Department of Primary Industries, Wollongbar, New South Wales, Australia; University of Cincinnati

**Keywords:** AMR, antimicrobial resistance, *Escherichia coli*, selective agar, surveillance, validation

## Abstract

Success in the global fight against antimicrobial resistance (AMR) is likely to improve if surveillance can be performed on an epidemiological scale. An approach based on agars with incorporated antimicrobials has enormous potential to achieve this. However, there is a need to identify the combinations of selective agars and key antimicrobials yielding the most accurate counts of susceptible and resistant organisms. A series of experiments involving 1,202 plates identified the best candidate combinations from six commercially available agars and five antimicrobials, using 18 Escherichia coli strains as either pure cultures or inocula-spiked feces. The effects of various design factors on colony counts were analyzed in generalized linear models. Without antimicrobials, Brilliance E. coli and CHROMagar ECC agars yielded 28.9% and 23.5% more colonies, respectively, than MacConkey agar. The order of superiority of agars remained unchanged when fecal samples with or without spiking of resistant E. coli strains were inoculated onto agars with or without specific antimicrobials. When antimicrobials were incorporated at various concentrations, it was revealed that ampicillin, tetracycline, and ciprofloxacin were suitable for incorporation into Brilliance and CHROMagar agars at all defined concentrations. Gentamicin was suitable for incorporation only at 8 and 16 μg/ml, while ceftiofur was suitable only at 1 μg/ml. CHROMagar extended-spectrum β-lactamase (ESBL) agar supported growth of a wider diversity of extended-spectrum-cephalosporin-resistant E. coli strains. The findings demonstrate the potential for agars with incorporated antimicrobials to be combined with laboratory-based robotics to deliver AMR surveillance on a vast scale with greater sensitivity of detection and strategic relevance.

**IMPORTANCE** Established models of surveillance for AMR in livestock typically have a low sampling intensity, which creates a tremendous barrier to understanding the variation of resistance among animal and food enterprises. However, developments in laboratory robotics now make it possible to rapidly and affordably process large volumes of samples. Combined with modern selective agars incorporating antimicrobials, this forms the basis of a novel surveillance process for identifying resistant bacteria by chromogenic reactions, including accurately detecting and quantifying the presence of bacteria even when they are present at low concentrations. Because Escherichia coli is a widely preferred indicator bacterium for AMR surveillance, this study identifies the optimal selective agar for quantifying resistant E. coli strains by assessing the growth performance on agars with antimicrobials. The findings are the first step toward exploiting laboratory robotics in an up-scaled approach to AMR surveillance in livestock, with wider adaptations in food, clinical microbiology, and public health.

## INTRODUCTION

Antimicrobial resistance (AMR) has been identified as one of the most serious threats to animal and human health in the current era (1). A key component for controlling AMR is the performance of surveillance to determine the prevalence and spread of resistant bacteria. The livestock sector has become a focus for surveillance because of the potential for AMR to transfer to humans along the food chain. Food products with a propensity to be contaminated with animal microflora, such as ground meat, are increasingly included in surveillance because of the risk of zoonotic pathogens undergoing selection for resistance in the animal gut or acquiring resistance via horizontal gene transfer (2–5). In both food and livestock, commensals such as Escherichia coli have been widely exploited for use in AMR surveillance, since they readily develop resistance during *in vivo* exposure to antimicrobials and are easily isolated as a ubiquitous component of the gut microflora (6). A barrier to improving surveillance in food and livestock is that the broth microdilution technique for evaluating antimicrobial susceptibility of bacterial colonies, as recommended by international reference organizations, is expensive and labor-intensive, although the process has been adapted well to a clinical context (7, 8). In national surveillance programs (such as the Danish Integrated Antimicrobial Resistance Monitoring and Research Program), sampling must typically be constrained due to the aforementioned drawbacks of the broth microdilution technique (9). For example, fewer than 300 commensal E. coli isolates are obtained from the same number of herds or flocks of a given animal species in a year, with food product surveys being similarly affected (9). The inferences that can be drawn from surveillance results are thus often constrained in scope and frequently fail to support decision making at the coalface of animal and food production, where changes to production management to control AMR arguably stand to have the greatest benefit. Therefore, an enhanced approach that can affordably assess a substantially larger number of isolates and samples within an authoritative design, to produce evidence on an epidemiological scale, rather than a clinical scale, is needed.

One way to achieve the scale described above is through large-scale enumeration of resistant E. coli strains from food or fecal samples using a process akin to the agar dilution technique for antimicrobial susceptibility testing (AST). Here, plating of diluted samples onto agars with incorporated antimicrobials is the foundation, which can be further automated using laboratory-based robotics (10). However, conventional solid agars used for AST, such as Mueller-Hinton agar (MHA), or traditional selective agars, such as MacConkey (MAC) agar, are unsuitable because they make it impossible to identify the target bacteria based solely on colony morphology and, especially in the case of MHA, the growth of nontarget bacteria is not adequately suppressed. Fortunately, modern selective agars for isolating E. coli are now commercially available. These agars suppress most nontarget organisms and achieve accurate colony identification using a chromogenic reaction (11). One key issue in the use of these agars is whether the activity of antimicrobials that are incorporated is compromised by other agar components, leading to inaccurate counts of resistant E. coli. A second key issue is whether the plating of diluted samples containing all of the microbial genera that are naturally occurring in the original samples (feces or food) interferes with the AST of the target organism (in this case, commensal E. coli). Previous studies showed that MAC agar with incorporated ciprofloxacin is able to selectively isolate ciprofloxacin-resistant E. coli strains (12–14), although tetracycline cannot be used with MAC agar in this way due to interference with antimicrobial activity by divalent cations (calcium and magnesium salts are integral components of MAC agar) (15–19). Similarly, there is a need to evaluate the suitability of commercial selective agars targeting extended-spectrum-cephalosporin (ESC)-resistant E. coli strains, such as Brilliance extended-spectrum β-lactamase (ESBL) and CHROMagar ESBL agars, for detection and enumeration under the same conditions.

This study aims to address these issues through three objectives. The first objective is to compare E. coli colony counts on selective agars (Brilliance E. coli and CHROMagar ECC agars) to assess which has the best E. coli growth performance for accurate enumeration of E. coli colonies. The second objective is to identify which combinations of specific concentrations of antimicrobials (ampicillin, tetracycline, gentamicin, ciprofloxacin, and ceftiofur) and selective agars achieve the most accurate enumeration of resistant E. coli strains (this includes equivalent evaluation of commercial agars for isolation of ESC-resistant E. coli strains) via colony counts. The third objective is to assess whether the ability to detect and quantify resistance is reduced when the target organisms are comingled with natural flora present in fecal samples. Together, the findings will serve to identify the optimal selective agar for achieving large-scale detection and quantification of resistant E. coli strains in samples from the food chain using laboratory robotics.

## RESULTS

### Experiment A: comparison of E. coli growth on commercial E. coli-selective agars.

**FIG 1:**
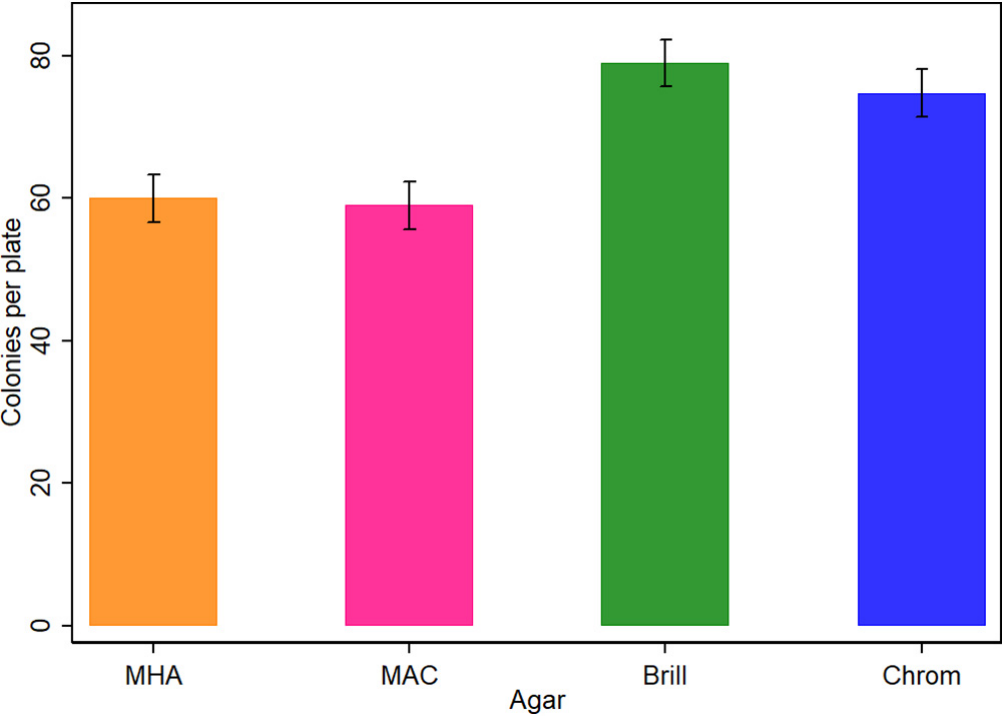
Comparisons of E. coli growth performance (mean colony counts per plate ± standard error [SE]) on three E. coli-selective agars and MHA (all without antimicrobials) (total number of plates, 160). The standardized inoculum across all agars consisted of diluted pure cultures of diverse E. coli strains. Means were calculated as marginal effects from linear model analysis.

Three selective agars and one nonselective agar without incorporation of antimicrobials were compared for the ability to support growth of diverse E. coli strains (with and without resistance to various antimicrobials) (see Table S1 at https://static1.squarespace.com/static/605d92065603b3328e679ddb/t/6177ab50c738d662e3b62344/1635232593287/Media+Validation+Supplementary+June+2021+AEM.pdf). All E. coli strains grew on agar without antimicrobials. Agar had a highly significant effect (*P* < 0.01) on colony growth, with the order of superiority being Brilliance agar (mean of 78.9 colonies per plate), CHROMagar agar (mean of 74.7 colonies per plate), MHA (mean of 60 colonies per plate), and MAC agar (mean of 59 colonies per plate) (Fig. 1). Although strain did have a significant effect on colony counts (*P* < 0.001), it did not change the aforementioned order of superiority of agars for any strain (see Fig. S1 at https://static1.squarespace.com/static/605d92065603b3328e679ddb/t/6177ab50c738d662e3b62344/1635232593287/Media+Validation+Supplementary+June+2021+AEM.pdf). In summary, E. coli counts on Brilliance agar, CHROMagar agar, and MHA were on average 28.9%, 23.5%, and 1.68%, respectively, higher than those on MAC agar (the worst performing).

All E. coli strains that were susceptible to ampicillin, tetracycline, ciprofloxacin, and ceftiofur did not grow on agars with the corresponding incorporated antimicrobial (at any concentration). However, E. coli strains that were susceptible to gentamicin grew on MAC (2 and 4 μg/ml), Brilliance (2 μg/ml), and CHROMagar (2 μg/ml) agars with incorporated gentamicin. All E. coli strains that were resistant to ampicillin, tetracycline, and ciprofloxacin grew on all agars with the corresponding incorporated antimicrobial (at any concentration). SA1001 was the only gentamicin-resistant E. coli strain that grew on all agars with incorporated gentamicin at all concentrations, while growth of SA44 (also resistant to gentamicin) was not observed on MHA with 8 and 16 μg/ml gentamicin incorporated. Ceftiofur-resistant E. coli strains grew on MAC agar with incorporated ceftiofur (at any concentration). In contrast, growth was inconsistent on Brilliance and CHROMagar agars when incorporated ceftiofur concentrations rose above 1 μg/ml (see Fig. S2 at https://static1.squarespace.com/static/605d92065603b3328e679ddb/t/6177ab50c738d662e3b62344/1635232593287/Media+Validation+Supplementary+June+2021+AEM.pdf).

**FIG 2:**
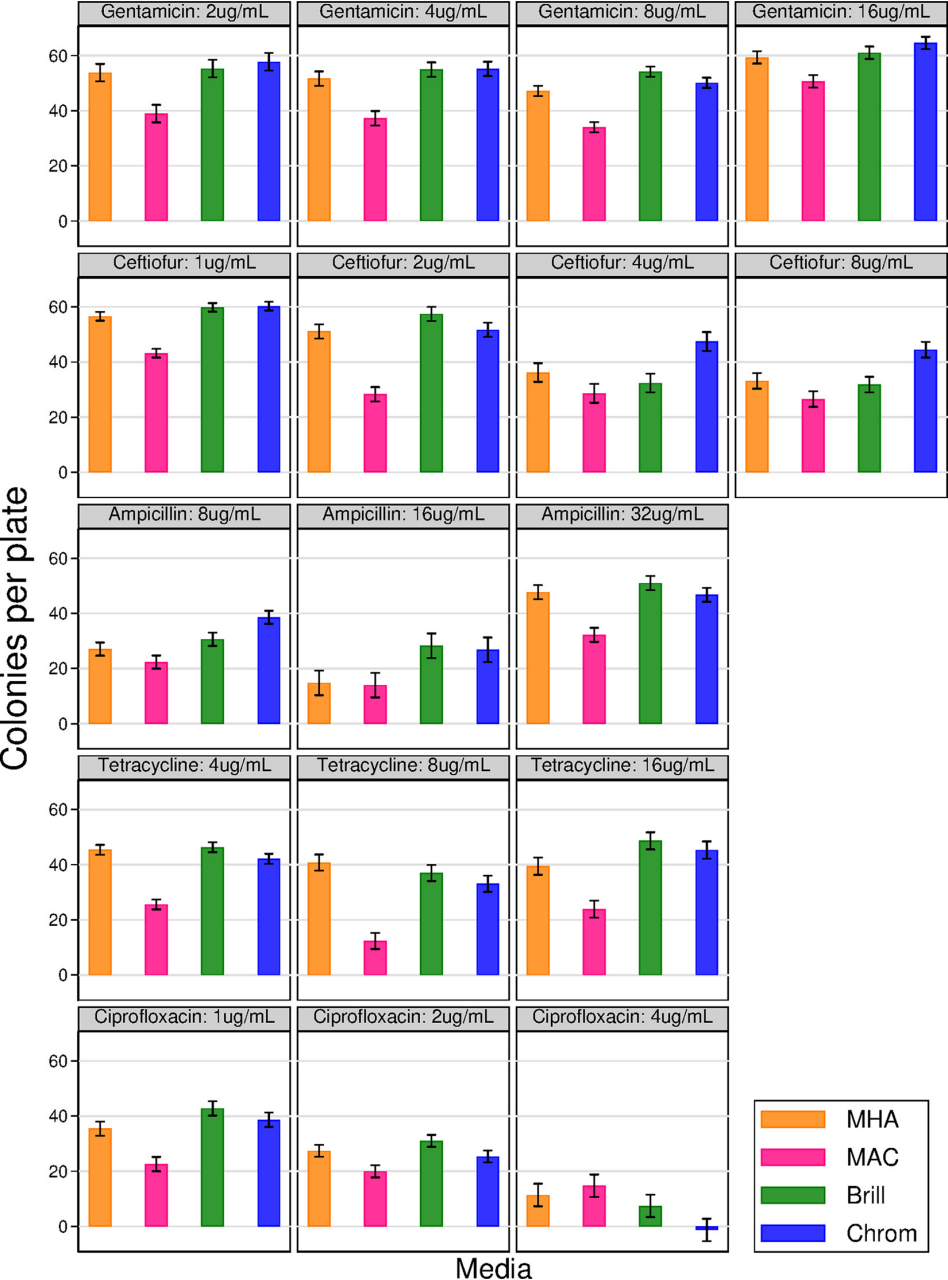
Comparisons of E. coli growth performance (mean colony counts per plate ± SE) on three E. coli-selective agars and MHA, each with incorporated ampicillin, tetracycline, gentamicin, ciprofloxacin, or ceftiofur at three or four concentrations (total number of plates, 424). The standardized inoculum across all agars consisted of diluted pure cultures of diverse E. coli strains resistant to each antimicrobial. Means were calculated as marginal effects from linear model analysis.

Separate linear models were constructed for each antimicrobial used. As with agar without antimicrobials, Brilliance and CHROMagar agars performed consistently better than MAC agar (Fig. 2); this includes Brilliance and CHROMagar agars with incorporated ceftiofur, which was superior to MAC agar with incorporated ceftiofur (Fig. 2). Antimicrobial concentration was found to have a significant effect for all antimicrobials tested (*P* < 0.001). Agar had a significant effect on all antimicrobials except tetracycline (*P* < 0.05), and strain had significant effects on all except tetracycline and gentamicin (*P* < 0.01). Significant interaction effects between strain and agar were found for tetracycline, gentamicin, and ceftiofur (*P* < 0.05), between agar and antimicrobial concentration for tetracycline (*P* < 0.01) and ceftiofur (*P* < 0.001), between strain and antimicrobial concentration for tetracycline and gentamicin (*P* < 0.05), and between all three factors for gentamicin (*P* < 0.01).

Finally, the three E. coli-selective agars, with and without incorporation of antimicrobials, were further tested using homogenized bovine fecal samples (with and without spiking of two fluoroquinolone [FQ]-resistant E. coli strains) (see Table S2 at https://static1.squarespace.com/static/605d92065603b3328e679ddb/t/6177ab50c738d662e3b62344/1635232593287/Media+Validation+Supplementary+June+2021+AEM.pdf). For agars without antimicrobials, the order of superiority was CHROMagar (mean of 35.6 colonies per plate), Brilliance (mean of 34.2 colonies per plate), and MAC (mean of 29.1 colonies per plate) agars (Table 1). For agars with incorporated ciprofloxacin, growth of FQ-resistant E. coli strains was observed on all agars regardless of bacterial concentration, and the order of superiority was Brilliance (mean of 32.8 colonies per plate), CHROMagar (mean of 28.3 colonies per plate), and MAC (mean of 22.8 colonies per plate) agars (Table 1). In this model, agar (*P* < 0.001), strain (*P* < 0.05), bacterial concentration (*P* < 0.001), and interactions between agar and bacterial concentration (*P* < 0.001) had significant effects.

**TABLE 1:** Comparisons of E. coli and FQ-resistant E. coli growth performance on E. coli-selective agars with and without incorporation of ciprofloxacin (total number of plates, 288), using homogenized bovine fecal samples with and without spiking of FQ-resistant E. coli strains

Homogenized bovine fecal sample	Growth (mean colony count/plate) on:					
	MAC agar		Brilliance agar		CHROMagar agar	
	Without antimicrobials	With ciprofloxacin	Without antimicrobials	With ciprofloxacin	Without antimicrobials	With ciprofloxacin
Without spiking^*a*^	29.1		34.2		35.6	
Spiked with FQ-resistant E. coli strains^*b*^		22.8		28.3		32.8

a Samples were not spiked with FQ-resistant E. coli and thus were inoculated only onto agars without ciprofloxacin.

b Samples were spiked with FQ-resistant E. coli and were inoculated only onto agars with incorporated ciprofloxacin.

### Experiment B: comparison of ESC-resistant E. coli growth on commercial ESC-resistant E. coli-selective agars.

**FIG 3:**
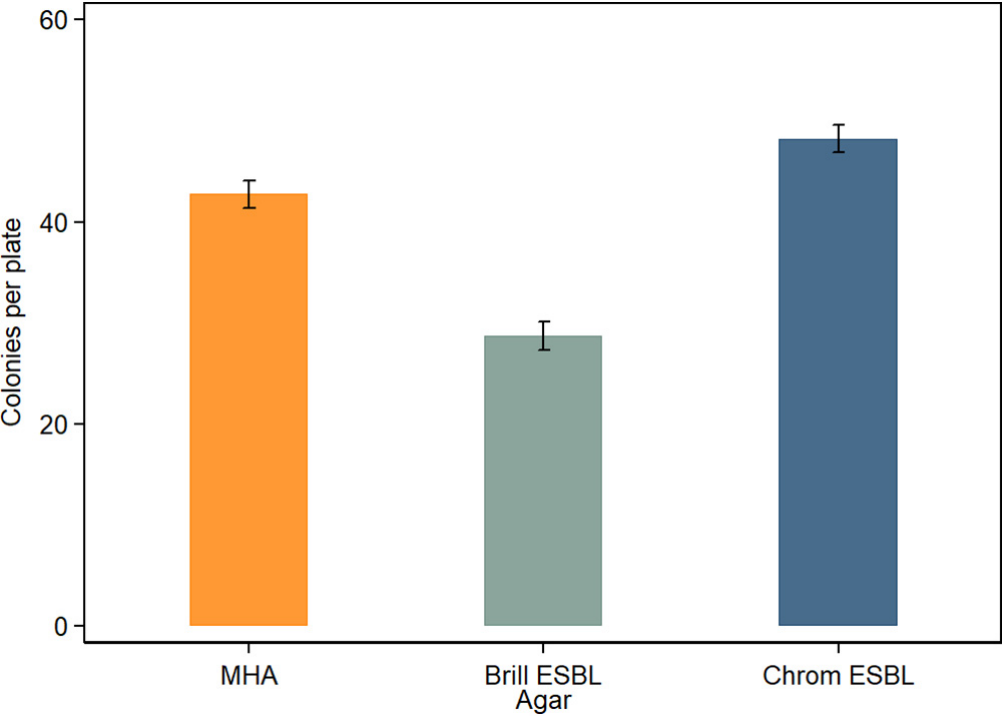
Comparisons of ESC-resistant E. coli growth performance (mean colony counts per plate ± SE) on two ESC-resistant E. coli-selective agars, with MHA (without antimicrobials) present as a control agar (total number of plates, 150). The standardized inoculum across all agars consisted of diluted pure cultures of diverse ESC-resistant E. coli strains. Means were calculated as marginal effects from linear model analysis.

Two ESC-resistant E. coli-selective agars (Brilliance ESBL and CHROMagar ESBL agars) were compared for the ability to support growth of diverse ESC-resistant E. coli strains (see Table S2 at https://static1.squarespace.com/static/605d92065603b3328e679ddb/t/6177ab50c738d662e3b62344/1635232593287/Media+Validation+Supplementary+June+2021+AEM.pdf). The nonselective MHA without antimicrobials was used as a control agar. All ESC-resistant E. coli strains grew on all agars with the exception of SA27, which did not grow on Brilliance ESBL agar. CHROMagar ESBL agar (mean of 48.24 colonies per plate) best supported growth, followed by MHA (mean of 42.72 colonies per plate) and Brilliance ESBL agar (mean of 28.74 colonies per plate) (Fig. 3), and this order of superiority was also observed for each ESC-resistant E. coli strain (see Fig. S3 at https://static1.squarespace.com/static/605d92065603b3328e679ddb/t/6177ab50c738d662e3b62344/1635232593287/Media+Validation+Supplementary+June+2021+AEM.pdf). In this model, all factors and their associated interactions had significant effects (*P* < 0.001) on colony counts.

**FIG 4:**
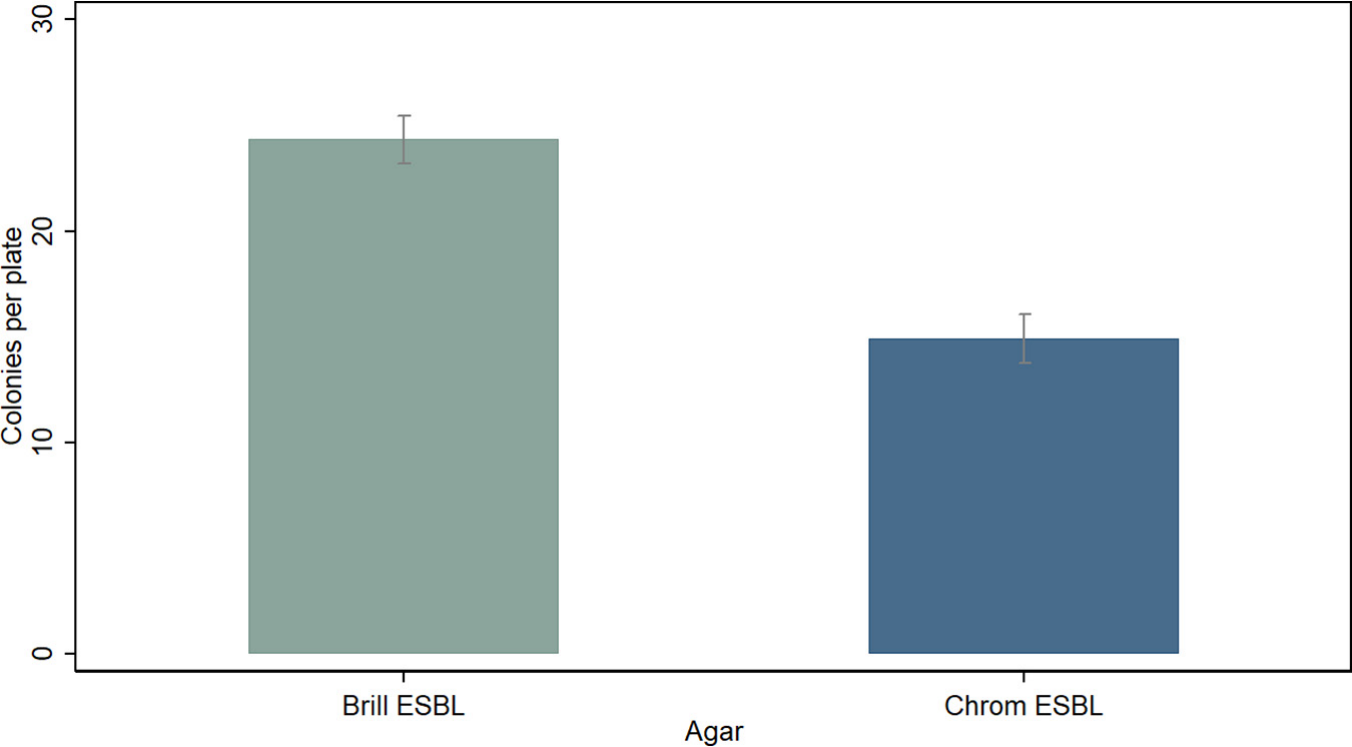
Comparisons of ESC-resistant E. coli growth performance (mean colony counts per plate ± SE) on two ESC-resistant E. coli-selective agars (total number of plates, 180). The standardized inoculum across all agars consisted of homogenized bovine fecal samples spiked with diverse ESC-resistant E. coli strains. Means were calculated as marginal effects from linear model analysis.

Finally, Brilliance ESBL and CHROMagar ESBL agars were further tested using homogenized bovine fecal samples spiked with 10 ESC-resistant E. coli strains (see Table S2 at https://static1.squarespace.com/static/605d92065603b3328e679ddb/t/6177ab50c738d662e3b62344/1635232593287/Media+Validation+Supplementary+June+2021+AEM.pdf). Brilliance ESBL agar (mean of 24.3 colonies per plate) was found to be superior to CHROMagar ESBL agar (mean of 14.9 colonies per plate) (Fig. 4), with the same superiority order observed for each ESC-resistant E. coli strain (see Fig. S4 at https://static1.squarespace.com/static/605d92065603b3328e679ddb/t/6177ab50c738d662e3b62344/1635232593287/Media+Validation+Supplementary+June+2021+AEM.pdf). The only exception was SA27, which did not grow on Brilliance ESBL agar regardless of bacterial concentration. All factors, including associated interactions, had significant effects (*P* < 0.001) on colony counts.

## DISCUSSION

AMR surveillance in livestock and food products is a critical tool for progressive antimicrobial stewardship, prevention of AMR spread, and the preservation of effective antimicrobials. Through the combination of high-throughput robotics and selective agar with the antimicrobial of interest incorporated, it is possible to quantify carriage levels and prevalence of resistance. With E. coli being used as a common indicator bacterium in AMR surveillance systems (6), this study aimed to identify the optimal selective agar and antimicrobial concentrations for quantifying resistant E. coli populations for AMR surveillance in livestock.

In this study, three selective agars were tested (MAC, Brilliance and CHROMagar agars). Despite the presence of other experimental factors and interactions significantly affecting colony counts, Brilliance and CHROMagar agars were comparable in performance and were consistently superior to MAC agar in all situations (pure cultures, fecal samples, and fecal samples spiked with FQ-resistant E. coli strains), as demonstrated through a greater number of E. coli colonies. The superior growth performance on Brilliance and CHROMagar agars can be attributed to the basic function and design of the agars. Both selective agars were specifically formulated for growing coliform bacteria and, while the exact ingredients in the selective mixes of the two agars are not disclosed by the manufacturers, there may be components that provide specific growth support for coliform bacteria, including E. coli. In contrast, the consistently inferior performance of MAC agar could be attributed to its components, which indiscriminately select for Gram-negative bacteria. Unlike Brilliance and CHROMagar agars, MAC agar possesses bile salts as its selective component to suppress Gram-positive bacterial growth through induction of DNA damage (20). However, it is likely that this bile salt mechanism also indirectly exerts a suppressive effect on E. coli growth, due to E. coli constantly having to express genes that reduce the growth rate in order to repair any DNA damage (21). Therefore, the indiscriminate selection combined with the suppressive effect of bile salts in MAC agar presents a more stressful environment for E. coli, resulting in inferior performance. Additionally, the consistent performance of Brilliance and CHROMagar agars demonstrated that the ability of the two agars to support susceptible and FQ-resistant E. coli growth for detection and quantification was not impeded by the copresence of fecal microflora.

Ampicillin, tetracycline, gentamicin, ciprofloxacin, and ceftiofur were incorporated into each agar to identify the best concentration for growing the corresponding resistant E. coli strain for quantification. MAC agar consistently supported less growth regardless of antimicrobial and concentration; therefore, it is not considered appropriate for quantitative AMR surveillance. Only ampicillin, tetracycline, and ciprofloxacin were found to be suitable for incorporation into Brilliance and CHROMagar agars at all defined concentrations, with growth of all resistant strains being observed. In contrast, gentamicin was suitable for incorporation into Brilliance and CHROMagar agars only at 8 and 16 μg/ml, as growth of susceptible strains were observed at lower concentrations. A higher number of susceptible strains grew on CHROMagar agar than on Brilliance agar, which suggests a higher level of suppression of gentamicin activity with the former. Currently, it is difficult to ascertain the mechanism by which this suppression occurs, although one possibility could be the significant three-way interaction between all factors.

A significant interaction between agars and ceftiofur was identified, which, given the unexpected growth inhibition of some ceftiofur-resistant E. coli strains at higher ceftiofur concentrations, indicates a likely synergistic effect of ceftiofur with agar, resulting in greater ceftiofur activity. It is possible that this synergy also extends to 1 μg/ml, despite all ceftiofur-resistant E. coli strains growing at this concentration. With the lack of information in the current literature pertaining to interactions between agar and ceftiofur, further investigation is needed to explain this phenomenon. Nonetheless, this indicates that ceftiofur is not suitable for incorporation into Brilliance and CHROMagar agars, and we suggest that either ESC-resistant E. coli-selective agar, such as CHROMagar ESBL agar, be used for quantitative AMR surveillance of ESC-resistant E. coli strains or further investigation into the viability of using other third-generation cephalosporin antimicrobials, such as cefotaxime or ceftriaxone, for incorporation into selective agar be performed.

Finally, both Brilliance ESBL and CHROMagar ESBL agars had unique advantages. While Brilliance ESBL agar was superior in supporting growth of ESC-resistant E. coli strains from spiked homogenized fecal samples, CHROMagar ESBL agar was able to support a wider diversity of ESC-resistant E. coli strains. This was evident from the absence of SA27 growth on Brilliance agar, in contrast to its growth on CHROMagar agar, regardless of whether it was from a pure culture or a spiked homogenized fecal sample, which also serves to demonstrate that the interference with SA27 (and thus ESC-resistant E. coli) growth on both agars is likely due to interactions between strain and agar, rather than the copresence of fecal microflora. Nevertheless, the ability of CHROMagar ESBL agar to capture a wider diversity of ESC-resistant E. coli strains makes it better suited for AMR surveillance than Brilliance ESBL agar, as it would increase the probability of detecting ESC-resistant E. coli strains.

The reason for growth variation between strains was not clear, as it was not the principal feature being evaluated. Most data in the current literature focus on growth rates of E. coli strains under specific environmental conditions, but no studies have evaluated possible factors influencing growth rates among E. coli strains (22–24). Significant interaction of strain with agar or antimicrobial is one such factor affecting growth rates, but the uniformity in performance across all agars, with and without incorporation of antimicrobials, suggests that this influence on growth was minimal and not enough to affect the performance outcome for each agar.

This study represents the first step toward establishing an enhanced AMR surveillance approach for assessing AMR in livestock and food products. In contrast to the established approach of AMR surveillance, this enhanced approach is both qualitative and quantitative in nature and is built on the capacity to rapidly identify E. coli colonies on agars for colony enumeration. When combined with robotics, it provides exciting opportunities for up-scaling based on programming and machine learning pathways to allow the identification of E. coli colonies based on colony color for enumeration, with reduced human input and potentially greater accuracy (10). The practical ramifications of this are that more accurate information can be obtained from a greater number of samples, which increases the sensitivity of detecting a given phenotype across a population of animals and herds. It is an especially relevant technique for early detection of resistance to critically important antimicrobials (CIAs), since it cannot be assumed either that the level of colonization is uniform across animals or herds (25) or that the phenotypes of interest are present at high enough concentrations to be found by traditional AST means. Moreover, any positive colonies detected can be preserved for genomic interrogation to understand their ecological origins, as demonstrated in studies of human-wildlife-livestock transmission (26).

Based on this study, we recommend the use of Brilliance and CHROMagar agars with and without incorporation of antimicrobials, as well as CHROMagar ESBL agar. in combination with robotics to evaluate the feasibility of this enhanced approach. Additionally, this enhanced approach has promising applications within food, clinical, and public health settings, through large-scale qualitative and quantitative AMR surveillance of critically important antimicrobial-resistant bacteria to support infection control and evaluation of the effectiveness of antimicrobial stewardship (27).

## MATERIALS AND METHODS

All agars used in this study were commercially available and were used as directed by the manufacturer, with the exception of the incorporation of additional antimicrobials as demanded by the study design.

### Experiment A: comparison of E. coli-selective agars with and without incorporation of antimicrobials.

**FIG 5:**
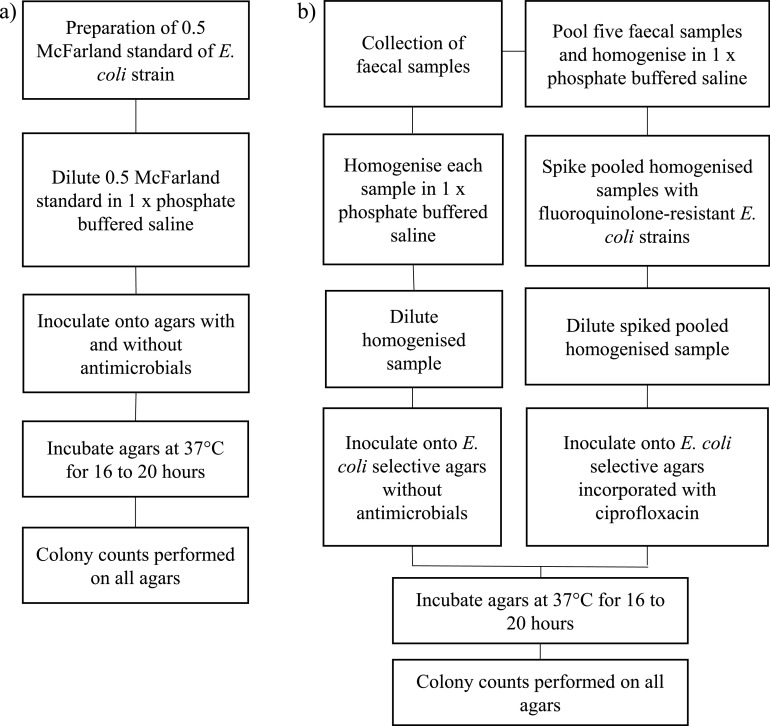
Overview of the general procedure for experiment A. (a) Procedure using pure cultures of E. coli strains. (b) Procedure using homogenized fecal samples with and without spiking of FQ-resistant E. coli strains.

The performances of growing E. coli on three E. coli-selective agars and a fourth nonselective control agar, with and without incorporation of antimicrobials, were compared using pure cultures of diverse E. coli strains, with an overview of the general procedure shown in Fig. 5a. All agars without antimicrobials were purchased directly from suppliers. The three selective agars used were MAC (Edwards Group), Brilliance (Thermo Fisher Scientific), and CHROMagar (MicroMedia, Edwards Group) agars. MHA (Edwards Group) was used as the fourth agar and was chosen for comparison due to its status as the gold standard nonselective agar for routine AST (28). The same four agars with incorporated antimicrobials were prepared in-house using the agar dilution technique, according to the manufacturer’s instructions. Both Mueller-Hinton broth powder (Oxoid, Thermo Fisher Scientific) and agar number 1 powder (Oxoid, Thermo Fisher Scientific) were used to prepare MHA. MacConkey number 3 powder (Oxoid, Thermo Fisher Scientific) was used to prepare MAC agar. Brilliance agar was prepared using Brilliance E. coli/coliform-selective medium powder (Oxoid, Thermo Fisher Scientific), while E. coli-coliforms chromogenic medium (Conda, Edwards Group) was used to prepare CHROMagar agar. The antimicrobials selected for incorporation into agars were ampicillin, tetracycline, gentamicin, ciprofloxacin, and ceftiofur (from the penicillin, tetracycline, aminoglycoside, FQ, and third-generation cephalosporin families, respectively). These were included due to their importance in the livestock and public health sectors (particularly ciprofloxacin and ceftiofur, which are CIAs for human medicine); they are often included in AMR surveillance programs involving livestock and food products (3, 5, 29–32). All antimicrobial stocks were prepared using antimicrobial powders (Sigma-Aldrich) and were stored following Clinical and Laboratory Standards Institute (CLSI) guidelines (28). All stocks were used within the shelf life detailed by the manufacturer. Prior to pouring of the agars into petri dishes, antimicrobials were added to sterilized agars after cooling in a 60°C water bath, to obtain specific concentrations for each antimicrobial. Three concentrations were chosen for ampicillin (8, 16, and 32 μg/ml), tetracycline (4, 8, and 16 μg/ml), and ciprofloxacin (1, 2, and 4 μg/ml), while four were chosen for gentamicin (2, 4, 8, and 16 μg/ml) and ceftiofur (1, 2, 4, and 8 μg/ml). All concentrations were chosen to cover the clinical breakpoints for E. coli as listed by the CLSI, with the epidemiological cutoff points (ECOFF) listed by the European Committee on Antimicrobial Susceptibility Testing (EUCAST) covered for ampicillin, tetracycline, gentamicin, and ceftiofur (33, 34). After the addition of antimicrobials, 20 ml of the agar mixture was poured into 90-mm-diameter circular petri dishes and left to solidify under a laminar flow hood. All agars with incorporated antimicrobials were stored in the dark at 4°C and used within 2 weeks after preparation.

Eight E. coli strains were chosen; ATCC 25922 was included as the quality control strain, while the remaining seven strains were E. coli strains isolated from different animal species. SA44 was isolated from pigs (35), SA1000, SA1001, and SA1002 were isolated from Australian silver gulls (26), and SA1003, SA1004, and SA1005 were archival in-house strains (see Table S1 at https://static1.squarespace.com/static/605d92065603b3328e679ddb/t/6177ab50c738d662e3b62344/1635232593287/Media+Validation+Supplementary+June+2021+AEM.pdf). The rationale for the selection of these strains was to achieve diversity in origin to capture variations potentially present in wild-type populations of E. coli. Prior to the commencement of the experiment, MIC testing using the broth microdilution method was performed for all E. coli strains according to CLSI guidelines, to obtain the phenotypic resistance profile of each strain (28) (see Table S1 at https://static1.squarespace.com/static/605d92065603b3328e679ddb/t/6177ab50c738d662e3b62344/1635232593287/Media+Validation+Supplementary+June+2021+AEM.pdf). All growth observations on agars with incorporated antimicrobials were compared to the phenotypic resistance profile shown for each strain, in order to determine any unexpected absence or presence of E. coli growth. After overnight growth on Columbia sheep blood agar (Edwards Group), a suspension of each E. coli strain meeting the 0.5 McFarland standard was prepared using a nephelometer (Sensititre). Each standardized inoculum underwent 10-fold serial dilution to 10^−5^ in sterile 1× phosphate-buffered saline (PBS). Inoculation was performed by dispensing 80 μl of the 10^−5^ inoculum onto agar without antimicrobials and spreading it evenly across the agar surface using a sterile loop. Inoculation on agars without antimicrobials was repeated for a total of five replicates per combination of agar and strain, while inoculation on agars with incorporated antimicrobials was repeated for a total of two replicates per combination of agar, strain, and antimicrobial concentration. All agars were incubated for 16 to 20 h at 37°C. Presumptive identification of E. coli on Brilliance and CHROMagar agars was performed based on colony color, as detailed by the manufacturers. For MAC agar, pink colonies were presumed to be E. coli because most E. coli strains are known to be lactose fermenters. Because it is a nonselective agar, E. coli colonies on MHA appear colorless.

Homogenized bovine fecal samples were used as field samples to verify the performance for growing E. coli on the same three E. coli-selective agars without antimicrobials, with an overview of the general procedure shown in Fig. 5b. All agars without antimicrobials were purchased from the same suppliers as described above. Twenty bovine fecal samples from the Murdoch University farm were sampled. All fecal samples were collected from fresh fecal piles and processed on the day of collection. Approximately 2 g of each fecal sample was homogenized for 30 s in 18 ml of sterile 1× PBS using a BagMixer 400 P laboratory blender (Interscience, Edwards Group). This was repeated two more times to obtain a total of three replicates per sample. The homogenized mixture of each replicate underwent 10-fold serial dilution, and 80 μl of each 10-fold dilution was inoculated onto each agar and spread evenly across the agar surface using a sterile loop. The procedures for agar incubation and presumptive identification of E. coli on agar were the same described above for agars without antimicrobials.

Homogenized bovine fecal samples spiked with FQ-resistant E. coli strains (see Table S2 at https://static1.squarespace.com/static/605d92065603b3328e679ddb/t/6177ab50c738d662e3b62344/1635232593287/Media+Validation+Supplementary+June+2021+AEM.pdf) were used as field samples to further evaluate the performance for growing FQ-resistant E. coli strains on the same three E. coli-selective agars with incorporated 4 μg/ml ciprofloxacin, with an overview of the general procedure shown in Fig. 5b. All agars were prepared in the same manner as described above for agars with incorporated antimicrobials. Sequence type 131 (ST131) and ST744 E. coli strains isolated from Australian silver gulls were chosen for inoculation into fecal samples due to their ubiquity as FQ-resistant E. coli strains internationally among both humans and animals (26, 36–39). The first 10 bovine fecal samples used previously were chosen for pooling. Each pooled sample consisted of five individual samples to form a total of two pooled samples. For each pooled sample, approximately 2 g of each individual fecal sample (total of approximately 10 g) was homogenized for 30 s in 90 ml of sterile 1× PBS using a BagMixer 400 P laboratory blender (Interscience, Edwards Group). This was repeated two more times to obtain a total of three replicates per pool sample. After overnight growth on Columbia sheep blood agar (Edwards Group), a suspension of each E. coli strain meeting the 0.5 McFarland standard was prepared using a nephelometer (Sensititre) and was inoculated into the homogenized mixture of each replicate to obtain bacterial concentrations of 10^3^, 10^5^, and 10^7^ CFU/g. Mixtures containing 10^5^ and 10^7^ CFU/g were serially diluted to 10^−1^ and 10^−3^ dilution factors, respectively. Eighty microliters of 10^3^, 10^5^, and 10^7^ at neat, 10^−1^, and 10^−3^ dilution factors, respectively, was inoculated onto each agar and spread evenly across the agar surface using a sterile loop. The procedures for agar incubation and presumptive identification of E. coli on agar were the same as described above for agars with incorporated antimicrobials. ATCC 25922 was also inoculated onto each agar as a quality control.

### Experiment B: comparison of ESC-resistant E. coli-selective agars.

**FIG 6:**
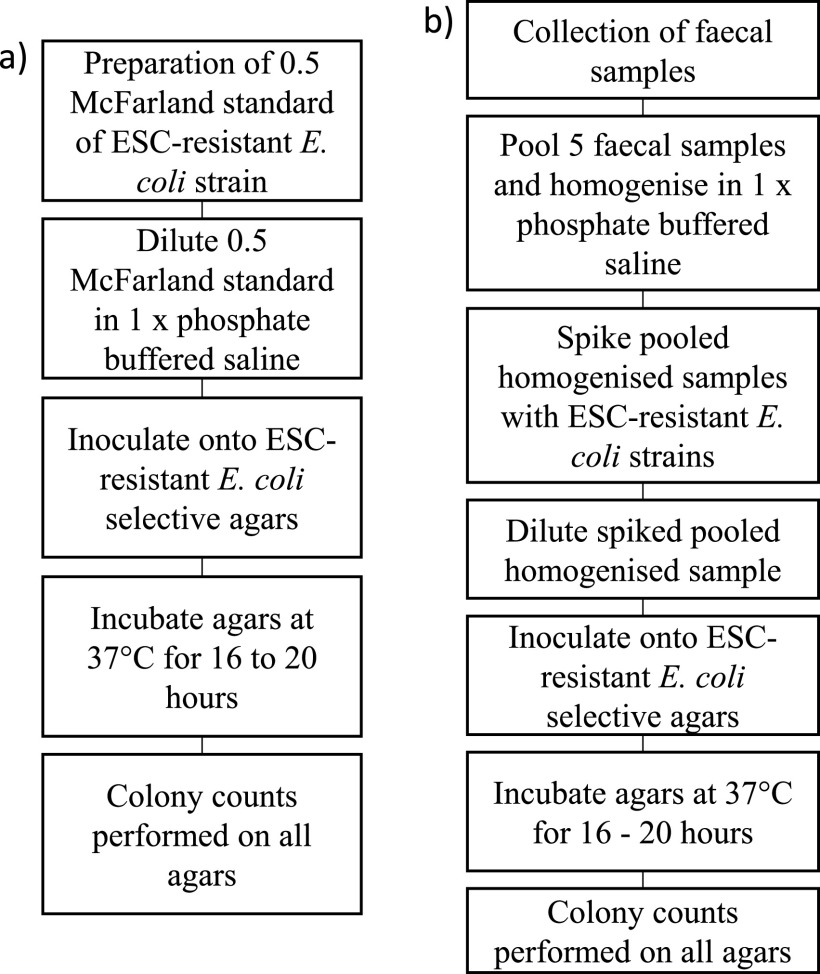
Overview of the general procedure for experiment B. (a) Procedure using pure cultures of ESC-resistant E. coli strains. (b) Procedure using homogenized fecal samples spiked with ESC-resistant E. coli strains.

The performances of growing ESC-resistant E. coli on two ESC-resistant E. coli-selective agars were compared using pure cultures of diverse ESC-resistant E. coli strains (see Table S2 at https://static1.squarespace.com/static/605d92065603b3328e679ddb/t/6177ab50c738d662e3b62344/1635232593287/Media+Validation+Supplementary+June+2021+AEM.pdf), with an overview of the general procedure shown in Fig. 6a. All agars were purchased directly from suppliers. Brilliance ESBL (Thermo Fisher Scientific) and CHROMagar ESBL (MicroMedia, Edwards Group) agars were the two ESC-resistant E. coli-selective agars, while MHA (Thermo Fisher Scientific) was selected as the nonselective agar. The supplier for MHA in experiment B differed from that in experiment A; however, the formulation of the agar was the same. Ten ESC-resistant E. coli strains were chosen, with each strain harboring a different gene conferring resistance to ESCs, in order to encompass the wide genotypic variations present among ESC-resistant E. coli strains (see Table S2 at https://static1.squarespace.com/static/605d92065603b3328e679ddb/t/6177ab50c738d662e3b62344/1635232593287/Media+Validation+Supplementary+June+2021+AEM.pdf). SA44 and SA1001 were the only two strains from experiment A included in experiment B (see Table S2 at https://static1.squarespace.com/static/605d92065603b3328e679ddb/t/6177ab50c738d662e3b62344/1635232593287/Media+Validation+Supplementary+June+2021+AEM.pdf). Of the remaining eight strains, SA27 was isolated from pigs (36), while SA1074, SA1075, SA1076, SA1077, SA1078, SA1079, and SA1080 were isolated from Australian silver gulls (26) (see Table S2 at https://static1.squarespace.com/static/605d92065603b3328e679ddb/t/6177ab50c738d662e3b62344/1635232593287/Media+Validation+Supplementary+June+2021+AEM.pdf). The procedures for culturing ESC-resistant E. coli strains, McFarland standard preparation, agar inoculation (including replicate numbers) and incubation, and presumptive identification of E. coli on MHA were the same as in experiment A using pure cultures of E. coli strains. Presumptive identification of ESC-resistant E. coli on Brilliance ESBL and CHROMagar ESBL agars was performed based on colony color, as detailed by the manufacturers. ATCC 25922 was also inoculated onto each agar as a quality control.

Homogenized bovine fecal samples spiked with ESC-resistant E. coli strains (see Table S2 at https://static1.squarespace.com/static/605d92065603b3328e679ddb/t/6177ab50c738d662e3b62344/1635232593287/Media+Validation+Supplementary+June+2021+AEM.pdf) were used as field samples to verify the performance for growing ESC-resistant E. coli strains on the same two ESC-resistant E. coli-selective agars, with an overview of the general procedure shown in Fig. 6b. Ten ESC-resistant E. coli strains were also chosen; nine strains, SA27, SA44, SA1001, SA1074, SA1075, SA1076, SA1077, SA1079, and SA1080, were the same strains as described above, while the last strain, SA1083, was another strain previously isolated from Australian silver gulls (26) (see Table S2 at https://static1.squarespace.com/static/605d92065603b3328e679ddb/t/6177ab50c738d662e3b62344/1635232593287/Media+Validation+Supplementary+June+2021+AEM.pdf). The first five bovine fecal samples used previously in experiment A were chosen for pooling. The procedures for pooling, strain inoculation into the homogenized fecal mixture, agar inoculation (including replicate numbers), and incubation were the same as in experiment A using homogenized bovine fecal samples spiked with FQ-resistant E. coli strains on agars with incorporated ciprofloxacin, with the exception that only Brilliance ESBL and CHROMagar ESBL agars were used. Presumptive identification of ESC-resistant E. coli on agar was the same as described above. ATCC 25922 was also inoculated onto each agar as a quality control.

### Statistical analysis.

Statistical analysis used the linear model framework in Stata version 16.0 (Stata Corp., College Station, TX, USA). All analyses were fixed-effect models with the count of E. coli colonies on each plate as the outcome. Factors in each model were determined by the design of each experiment and included type of agar, strain of E. coli, concentration of antimicrobial, and their interactions. The results were derived as estimates of marginal effects and expressed (in text and figures) as the mean effect of each combination of agar and antimicrobial concentration adjusted for E. coli strains used in the particular experiment and interaction terms. For experiments based on pure cultures of E. coli strains, a model was constructed for agars without antimicrobials and one model for each antimicrobial incorporated into agars. In the latter case, only E. coli strains resistant to the antimicrobial being evaluated were included in the linear model. For experiments based on fecal samples spiked with a mixture of E. coli strains, the analysis was similar, although the factor representing E. coli strain was not required.
